# Respiratory Microbiome of New-Born Infants

**DOI:** 10.3389/fped.2016.00010

**Published:** 2016-02-23

**Authors:** David J. Gallacher, Sailesh Kotecha

**Affiliations:** ^1^Department of Child Health, School of Medicine, Cardiff University, Cardiff, UK

**Keywords:** bronchopulmonary dysplasia, chronic lung disease, prematurity, microbiome, lung, colonization, 16S RNA gene, neonate

## Abstract

The respiratory tract, once believed to be sterile, harbors diverse bacterial communities. The role of microorganisms within health and disease is slowly being unraveled. Evidence points to the neonatal period as a critical time for establishing stable bacterial communities and influencing immune responses important for long-term respiratory health. This review summarizes the evidence of early airway and lung bacterial colonization and the role the microbiome has on respiratory health in the short and long term. The challenges of neonatal respiratory microbiome studies and future research directions are also discussed.

## Introduction

Respiratory insufficiency is the major issue limiting viability at the extremes of prematurity. Chronic lung disease of prematurity (CLD), also known as bronchopulmonary dysplasia, is the most common long-term complication of prematurity (1) with reduced lung function persisting into adulthood (2). Many risk factors have been identified in the development of CLD, including prematurity, supplemental oxygen therapy, mechanical ventilation, and patent ductus arteriosus (3). Systemic and pulmonary infections may also contribute to the development of CLD (4, 5). Despite the routine use of antenatal corticosteroids and exogenous surfactant, improved nutrition and ventilatory strategies, CLD remains a significant burden for surviving preterm infants, and an on-going challenge for neonatologists (6).

The microbiome is defined as the whole habitat of microorganisms (or of their genes) and the surrounding environment, with the term microbiota more specifically referring to the community of microorganisms living within a particular environment (7). The diversity of bacterial communities has been linked to important health outcomes (8). Diversity is a measure of how much variety is present in a community of microorganisms, regardless of the identities of the organisms. Diversity is made up of richness, the number of different bacterial species present, and evenness, defined as the relative abundance of the various species within the bacterial community (9). The composition of bacterial communities changes over time. A stable microbiota is resistant to such changes. The concept of dysbiosis refers to a pattern of bacterial colonization predisposing to disease (10).

The human host has evolved in symbiosis with microorganisms, which colonize multiple body sites. The interactions between host and the microbiota are only now being understood with microbiome science demonstrating that bacterial communities living within a human host have an influence over a diverse range of diseases (11). Within neonatal medicine, most of this work has focused on the role of the gut microbiome in the pathogenesis of necrotizing enterocolitis (NEC) (12–14), with widespread use of probiotics, a clinical result of this work (15). Attention is now moving beyond the gut to other body sites to identify the role of microbes in other anatomical locations including the respiratory tract.

The traditional notion of the lungs of healthy individuals being free from bacterial colonization is now defunct (16, 17). The lungs were initially not included in the human microbiome project (18) due to perceived sterility. The sterile lung hypothesis was driven by culture-based studies, designed to identify specific pathogens, failing to detect the low levels of difficult to culture commensal organisms within the lower airways and lungs. Culture-independent techniques utilizing sequencing technology to identify bacterial DNA have led to the discovery of communities of microorganisms within the airways and lungs of healthy individuals (19, 20). The alterations of microbial communities within the airways and lungs in pulmonary disease, and therefore the potential for therapeutic intervention, are only beginning to be grasped. This review summarizes the current evidence surrounding the early colonization of the airways and lungs of infants, and the impact this may have on short- and long-term health consequences.

## Methodological Challenges

In addition to the ethical and practical challenges that complicate neonatal research, respiratory microbiome work has to overcome challenges of difficult sample collection, contamination, and the low bacterial load in the lungs.

Bacteria are able to colonize the full length of the airways, from the nostril to the alveoli. The changes in anatomy and environmental exposure along this length lead to the creation of many varied niches for bacterial colonization. Sampling of the proximal airways at any age is straight forward using nasal swabs or nasopharyngeal aspirates. The inaccessibility of the lungs poses significant challenges for researchers. Sampling from the lungs and distal airways in adults has involved using sputum or endoscopic bronchoalveolar lavage (19, 21, 22). In the neonatal population, the lower airways of intubated infants can be accessed using tracheal aspiration to sample the endotracheal tube and trachea (23, 24) or non-bronchoscopic bronchoalveolar lavage (NB-BAL) to sample more distal airways (25). All these techniques are easily contaminated by upper respiratory tract or nasopharyngeal organisms. Avoiding such contamination using diseased explanted lungs and lungs from organ donors for whom no recipient could be found have demonstrated bacterial communities within lung tissue of adults (26, 27). The presence of bacterial DNA within the lungs of even healthy adults is no longer disputed (19, 20).

The particular difficulties of sampling the lung microbiome in neonates results in published studies concentrating on the upper airway or on ventilated infants (23, 28, 29). While the upper airway bacterial communities are related to those in the distal airways (30), it seems that the nasopharynx cannot be used as proxy for lung colonization (26, 30). Within the neonatal population, no studies have thus far reported on the lung microbiome of healthy term-born infants. In neonatal research, the vaginally delivered term-born, exclusively breast fed infant is considered most likely to develop the optimal microbiome (31). Other groups are generally compared to this assumed optimal microbial pattern. The lack of data from the airways and lungs of such infants complicates the interpretation of many neonatal studies. Studies of the gut microbiome reveal that the composition of gut flora is unique to an individual and shows marked variety between individuals (32). A similar variability between individuals has also been observed in the upper airways of children (33, 34). The concept of a “normal” microbiome or “normal” colonization pattern may not exist and may be difficult to define. Patterns of colonization or presence of predominant organisms are, however, distinguishable within populations and maybe associated with favorable or unfavorable outcomes.

Culture-independent techniques utilize sequencing technologies to identify organisms by sequencing target genes. The most commonly used gene for bacterial species identification is the 16S ribosomal RNA gene. The difference in nucleotide sequence within the nine hypervariable regions of the gene are used to identify the bacterial species present within a sample using next generation sequencing (35). All bacterial DNA is identified, not only from resident bacteria but also from dead microbes unable to survive in the sampled niche; and DNA from contaminant organisms acquired at any stage during sample collection or analysis (26). DNA-based techniques may therefore overestimate the community of bacteria residing in a habitat. A transcriptomic approach, using RNA sequences rather than DNA to identify bacteria (36), overcomes the problem of non-viable bacteria, but not contamination. A further alternative to 16S sequencing is a metagenomic approach when all nucleotide sequences are identified rather than an individual gene (37). This provides a more rigorous bacterial identification.

Many respiratory samples, particularly those from neonates, generate a low biomass (38) with low bacterial loads close to the limit of detection of the assays designed to detect such organisms. Careful use of negative controls at each stage of the analysis process is important to ensure validity of the results (39). Contamination during sample collection can be assessed by comparing samples from the lower respiratory tract with samples taken concurrently from the upper respiratory tract. Identical patterns of colonization would imply contamination (38). DNA extraction kits and PCR reagents are also known to be contaminated with bacterial DNA (40). Studies using low biomass samples need to demonstrate thorough record keeping of the lot numbers of reagents used and analyze for contamination from laboratory sources.

Despite the challenges and limitations of studying the airway and lung microbiome, a significant body of work is available relevant to adults, with several comprehensive reviews published (16, 41). The extra difficulties of research in this area in the neonatal population result in fewer published studies, with varying methodology. The evidence relevant to the neonatal population is the focus of this review.

## Acquisition of Microbiome

### Timing of Colonization

The *in utero* environment is traditionally considered to be physiologically sterile. This assumption has been challenged with the finding of bacteria in the placenta (42), fetal membranes (43), and amniotic fluid (44) of healthy pregnancies. It remains unclear if there is a low level of bacterial colonization of the *in utero* fetus, but bacterial presence has been detected in the first passage of meconium from healthy term-born infants (45), previously thought to be sterile, and in cord blood samples (46). Airway colonization may therefore begin *in utero* in some cases, and this is more likely in those exposed to chorioamnionitis, known to be a significant risk factor for preterm delivery. Bacterial DNA was detected from a larger proportion of preterm lungs and/or gastric fluid within 24 h of birth if delivered due to prelabor premature rupture of membranes or spontaneous preterm labor compared to those delivered by cesarean section for maternal or fetal reasons (47).

Within the first 5 min following birth, microbiological communities can be detected within the oral cavity and nasopharynx of term newborns (48), suggesting that colonization of the upper airways has already commenced. Initial studies searching for the presence of bacterial DNA in the lower respiratory tract of intubated preterm babies showed the presence of a diverse array of bacteria within the first week of life (24). In intubated preterm infants, one study noted that only 2 of 10 tracheal aspirate samples taken at <72 h of age contained detectable bacterial DNA. At 7 days of age, all 10 tracheal aspirates from the same babies contained detectable bacterial DNA (49). In contrast, Lohmann et al. detected bacterial DNA in all tracheal aspirate samples taken immediately after intubation on day 1 of life from 25 preterm neonates ≤32 weeks gestation (23). It appears that the colonization of the airways begins very early in life, at or possibly even before delivery.

*Ureaplasma* species, implicated in preterm neonatal respiratory infection, have been detected by molecular methods in tracheal aspirates samples at 24 h of age (24). Using RNA-based methodology, another study demonstrated that these organisms are transcriptionally active within the lungs for at least 3 weeks after delivery in some preterm neonates (50).

Studies vary in estimating the time for a stable respiratory microbiota to be established. One study suggests that bacterial density in the nasopharynx of healthy infants increases throughout the first year of life. Reducing diversity over time resulted in stable bacterial communities being established by 1 year of age (51). However, another study did not report a change in bacterial load in the nasopharynx after 1 month of age, but a continual evolution of organisms throughout the first 2 years of life (28).

The timing of bacterial colonization of the neonate remains controversial but a sterile *in utero* environment can no longer be assumed. Stable airway communities are not established during the neonatal period.

### Airway Colonizing Organisms

The adult lung microbiome has been more widely studied using molecular-based techniques. In healthy adult lungs, the phyla Bacteroidetes and Firmicutes predominate (around 80%) with Proteobacteria making up around 10% of the lung microbiome (19, 20, 52). At a genus level, *Streptococcus* and *Veillonella* species are the predominant Firmicutes organisms, and *Prevotella* species make up the majority of Bacteroidetes. Within Proteobacteria; *Pseudomonas*, *Haemophilus*, and *Neisseria* species are most common (41). The presence of known lung pathogens within this list demonstrates that tight immunological control over the microbiome occurs, and challenges the traditional view that respiratory infections are environmentally acquired.

Only limited information is available regarding the initial colonizers of the airways in the early days of life. One study of 10 intubated preterm infants using culture-free methodology noted a dominant organism was present (>50% of total sequences) in 31 of 32 tracheal aspirate samples taken in the first month of life. The most common dominant genus was *Staphylococcus*, in 19 samples, 17 of these being coagulase negative staphylococci. *Ureaplasma* species were dominant in nine samples from six subjects. Other species that predominated in a single sample, all between 14 and 21 days of life were *Pseudomonas aeruginosa*, *Enterococcus faecalis*, and *Escherichia coli* (49). A similar finding was reported in a separate study using tracheal aspirates collected during the first week of life. The two most common organisms identified using the 16S RNA gene were *Staphylococcus haemolyticus* and *Staphylococcus epidermidis*, both coagulase negative staphylococci (24). A subsequent study also demonstrated the presence of a dominant organism in many tracheal aspirate samples but noted that species within the Proteobacteria phylum, mainly *Acinetobacter* species, were dominant most often in the first day of life (23). Taken together, this evidence suggests that in the first days of life a pioneering colonizer becomes established but the identity of the organism depends on initial exposure of the infant.

### Factors Affecting Airway Colonization

The constituents of the infant gut microbiota have been shown to have been strongly influenced by delivery mode, feeding choices, and the perinatal use of antibiotics (53). The use of drugs such as H2 blockers affecting gastric acid secretion also affect early colonization patterns within the gut (54) and may indirectly affect the lungs. Such factors can have sustained effects on the gut microbiota beyond the neonatal period, with differences between vaginally and cesarean-born infants detectable until 12 months of age (55).

Evidence from the airways is less substantial, but the nasopharynx of term-born infants share the same initial colonizing organisms as various skin sites and the mouth. All sites demonstrated a large influence of the delivery method (48). Infants born by normal vaginal delivery had bacterial communities resembling maternal vaginal flora, while infants born by cesarean section were colonized by maternal skin organisms.

Mechanically ventilated preterm infants exposed to chorioamnionitis appear to have decreased species diversity in their tracheal aspirates although the trend did not reach statistical significance compared to unexposed infants (23). This may reflect overgrowth of pathogenic species in infants exposed to chorioamnionitis. Bacterial load estimation was not made in this study. It would have been interesting to compare the number of organisms present between chorioamnionitis exposed and unexposed infants.

Antibiotic use is widespread in neonatal patients, particularly in those born preterm. Exposure to antibiotics has been shown to reduce diversity and modify colonization patterns of the neonatal gut (56). Antibiotics also induce a significant change in the microbiota of sputum of cystic fibrosis patients (57). The impact on the lungs of neonates is unknown; however, in the upper airways of infants under 12 months of age, those who had received antibiotics in the preceding 4 weeks displayed a reduced proportion of *Alloiococcus* and *Corynebacterium*, with an increased proportion of potential pathogens, including *Haemophilus*, *Streptococcus*, and *Moraxella* (58).

Over 200 species of bacteria have been isolated from human breast milk including the beneficial *Bifidobacterium* and *Lactobacillus* species (59). Breast milk has the ability not only to pass on desirable colonizing flora from mother to baby but also to provide oligosaccharides to promote development of healthy microbiota (60).

Evidence is divided on the factors affecting the bacterial colonization of the nasopharynx of infants under 12 months of age. One study reported that at 2 months of age, mode of delivery and feeding methods did not affect the composition of the nasopharyngeal microbiome; however, the presence of siblings, recent respiratory tract infections, and attending day care all made a significant difference (58). In contrast, a well-designed study comparing exclusively breast fed and exclusively formula fed infants showed detectable changes in the nasopharyngeal microbiota at 6 weeks of age (61). A similar study showed a significant seasonal effect with a bloom of *Corynebacteriaceae* in the summer months, but *Pasteurellaceae* predominated in the winter months (51). These differences may also be explained by geographical differences, with the studies conducted in the Netherlands, Switzerland, and Australia.

Evidence from patients with cystic fibrosis followed up to 21 months of age, suggests that species colonizing the upper-airway (oropharyngeal samples) are first present in the stool (62). This suggests that the airway microbiota may be established from that of the gut. In healthy adult subjects who underwent microbiome analysis from the oral cavity, nasal cavity, lungs and stomach, the lung microbiome closely resembled the microbiome of the oral cavity more than other sites (30). Micro-aspiration of saliva into the airway is known to occur during sleep in healthy individuals and may result in aspiration of physiologically significant numbers of bacteria (63).

Accurate determination of the source of the microbiota would require deep sequencing of the genome of organisms from multiple sites including maternal skin, breast milk, birth canal, and from the skin of midwifery or neonatal staff caring for the infant, as well as range of environmental sites – a major undertaking.

Evidence suggests that the airway microbiome is affected by multiple environmental factors. Interpreting the studies is difficult given the different sampling techniques and anatomical sites tested; however, evidence is consistent with studies of bowel colonization showing an effect of delivery mode, antibiotics, and feeding methods.

## Chronic Lung Disease of Prematurity

Dysbiosis is defined as an imbalance in the microbes present in a particular niche that contributes or predisposes to disease (10, 16). Adult-based studies have suggested that airway dysbiosis can predict poor outcomes in cystic fibrosis (64) and in asthma (65). It is still unclear if dysbiosis reflects a change in environmental conditions within the airways in those with more severe disease or if dysbiosis is driving the poor prognosis.

Preventing CLD is a significant challenge in neonatology. Pulmonary infection has been suspected as an important contributing factor to CLD (4, 5) with organisms such as *Ureaplasma* species repeatedly implicated (66). The role of the commensal bacteria is less well studied. Early work using culture independent techniques comparing infants with and without CLD showed that detectable bacterial DNA (16S gene) in NB-BAL fluid was associated with developing CLD. The risk was even higher if the bacteria were present within the first 3 days of life (4). Organisms associated with CLD in this study were *E. coli*, *Haemophilus influenzae*, *Enterobacter* species, and *P. aeruginosa*. Further work showed the presence bacterial DNA in NB-BAL fluid was associated with higher proteinase activity within the lung, and the chance of developing CLD. Predominant organisms identified with high proteinase activity in the CLD group were *Staphylococcus aureus* and *E. coli* (67). The proteinases analyzed in this study were neutrophil derived. A greater immune response due to the presence of bacteria could explain the increased proteinase activity and the mechanism of the bacteria influencing the pathogenesis of CLD. Bacterial proteinases have been implicated in the pathogenesis of gut pathologies (68). The much lower bacterial load in the lungs compared to the gut make the role of colonizing microorganism derived proteinases less likely to be significant in pulmonary diseases, but this remains uncertain.

Another study investigated the difference between airway microbiota in tracheal aspirates from preterm infants with and without CLD using culture-independent techniques (23). Day one colonization patterns were similar in both groups with the predominance of *Acinetobacter*. Those infants who developed CLD had reduced species diversity and higher proportions of the pathogens *Staphylococcus* and *Klebsiella*. Those infants who did not develop CLD showed more stable bacterial communities with greater diversity. This suggests a role for the microbiome in the development of CLD but correlation between pro-inflammatory cytokines and microbial colonization patterns was not witnessed. The microbiome is likely to influence the development of CLD by stimulating pro- or anti-inflammatory responses. The study, however, was not able to provide such a link. Without a plausible mechanism of effect, differences in the microbiome between those with and without CLD may reflect different environmental conditions within the airways of those predisposed to more severe disease due to an independent factor, rather than a causal relationship. Further work is clearly needed to corroborate this study, but the concept of diverse bacterial communities being advantageous follows the pattern of evidence with reports in other diseases including respiratory and gut pathologies (8, 69).

The influence of the microbiome over CLD has been the target of studies due to the chronic nature of CLD and the known impact of bacterial infection. No studies have looked at the impact of the microbiome on other respiratory conditions affecting the neonate.

## Microbiological Programing

The early programing theory suggests that early life exposure to microbes is important for long-term health. Epidemiological data suggest a period in infancy that can determine future respiratory health (70, 71). Early colonization patterns are thought to affect immune development and potentially prime the host for later disease. This process was demonstrated in the gut, where early microbial colonization can significantly impact the long-term risk of asthma and allergy (72, 73). The respiratory microbiota also has a role in programing. Infants who were shown to be colonized (culture positive from hypopharyngeal aspirate) at 4 weeks of age with one of *Streptococcus pneumoniae, H. influenzae*, and/or *Moraxella catarrhalis* (but not *S. aureus*) were at significantly higher risk of pneumonia and bronchiolitis by the age of 3 years (74). The same cohort was followed up again at 5 years of age and the same organisms were associated with an increased risk of asthma (75). Interestingly, microbial colonization status at 12 months of age was not correlated with outcomes. The early colonization was important suggesting not only that a critical phase in immune programing exists but also that there may be a window of opportunity early in life to cultivate a healthy or protective microbiome. Cytokine data from these patients showed that the T helper cell type induced by colonization was significantly different. *M. catarrhalis* and *H. influenzae* induced a mixed T helper cell response. *S. pneumoniae* colonization was not associated with a significant change in mucosal cytokines (76). These results demonstrate a possible mechanism of action for the influence of the bacteria over the immune system. By inducing a mixed T helper cell response, the Th1 responses needed for intracellular bacteria destruction may be impaired, potentially leading to chronic inflammation (76). These pathways provide potential therapeutic targets for intervention to influence this process pharmacologically.

An Australian study using culture independent techniques to identify bacterial and viral DNA within infants’ nasopharynx also showed that early (around 2 months of age) asymptomatic carriage of streptococcus species significantly increases the risk of chronic wheeze at 5 years of age (58). In a similar culture-independent study, sampling the nasopharyngeal microbiota repeatedly over the first 2 years of life, the early (6 weeks of age) microbiota composition determined the future stability of the microbiota. Stable profiles were characterized by the presence of *Moraxella* and *Corynebacterium* or *Dolosigranulum* and were associated with breast feeding. Less stable profiles were marked by high abundance of *Haemophilus* or *Streptococcus*. Parent-reported respiratory infections were reduced in the stable microbiota group (28).

The combined evidence suggests early colonization of the gut and the respiratory tract may have a role in determining future respiratory health. The factors previously discussed affecting the early colonization patterns may therefore have a lasting impact beyond the neonatal period. Further work to understand the mechanisms of microbiological programing may assist in developing novel treatments or reveal possible ways to influence the microbiota to promote long term health.

## Gut–Lung Axis

**Figure 1:**
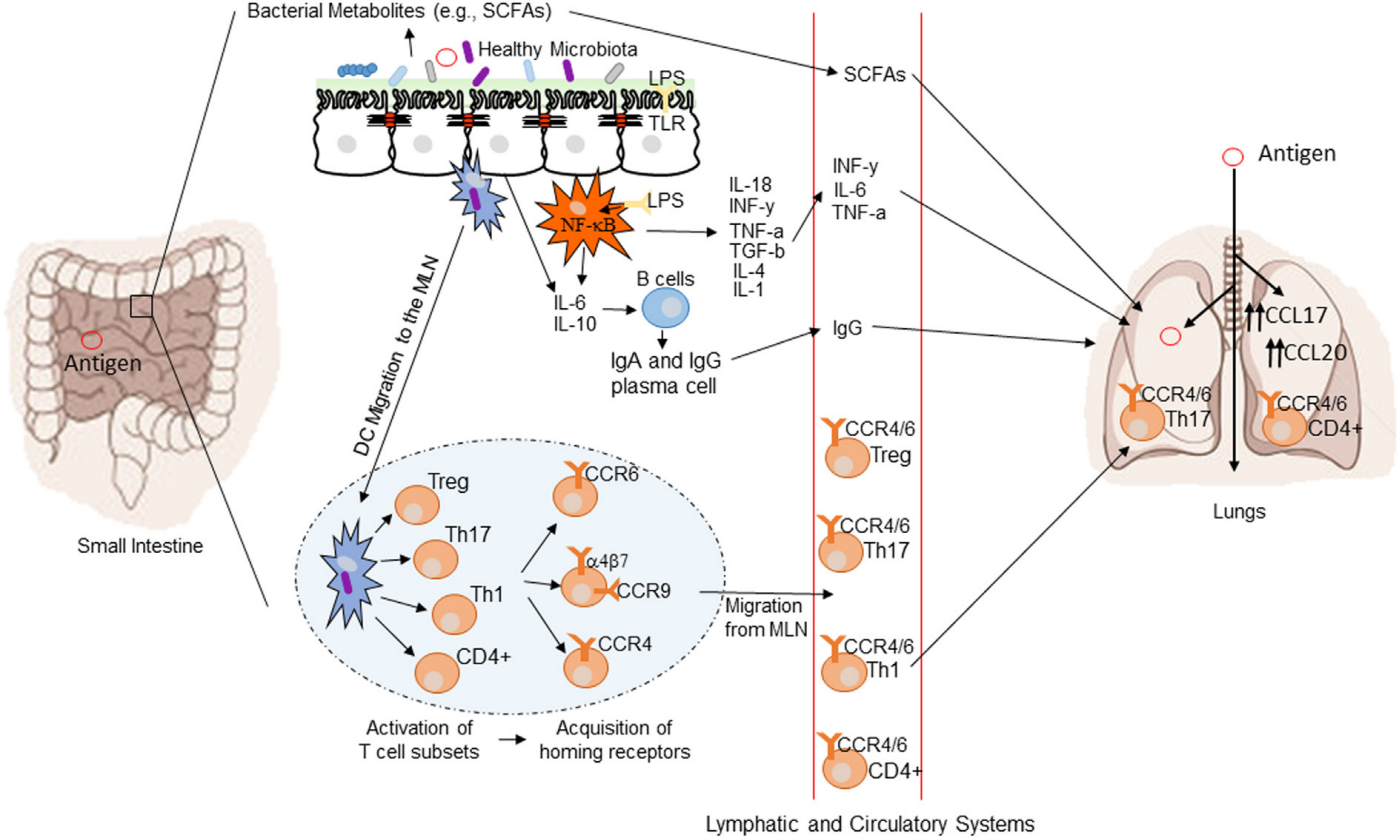
**Conceptual figure of the gut–lung axis**. Proposed model for the regulatory influence of the gastrointestinal microbiota on the immunology of the lung. Microbes in the intestine are sampled by dendritic cells (DCs) either directly from the lumen or following translocation through M cells to the gut-associated lymphoid tissue (GALT). A combination of signals from the microbes results in phenotypic changes in the DCs and migration to the draining lymph node. DCs promote the activation of various T cell subsets within the mesenteric lymph nodes (MLN) and the production of various regulatory cytokinesm, such as IL-10, TGF-b, INFg, and IL-6. T cell subsets then acquire immune homing molecules (i.e., CCR6, CCR4, and CCR9). Following immune challenge in the airway, cells activated in the GALT and MLN traffic to the respiratory mucosa *via* CCR4 or CCR6 where they promote protective and anti-inflammatory responses. Production of various bacterial metabolites (e.g., SCFAs) also affects the gut–lung axis, as these products are transported to the lung, where they can alter the levels of inflammation. Modified from Samuelson et al. (78).

The GI tract microbiome has the ability to influence diseases anatomically distant from the gut *via* immunological modulation of mucosal immune responses and the plethora of small metabolite molecules produced by bacteria (77). Figure 1 demonstrates a conceptual model of the mechanisms underlying the gut–lung axis (78). As previously mentioned, the additional influence of the gut over airway and lung bacterial colonization may also be extended to direct transfer of bacteria through micro-aspiration (63). H2 blockers or other drugs affecting the gastric acid secretion may affect lung colonization by affecting the composition of bacteria transferred by micro-aspiration (54). H2 blockers have been shown to be associated with the risk of late onset sepsis and NEC (79, 80) and in one study pneumonia (81) in preterm newborn infants. Thus, use of drugs, which may alter the microbiome, may have wider ranging consequences.

The gut microbiome may have a greater effect on respiratory health than previously appreciated (82). Clinical evidence corroborates the presence of the gut–lung axis. Oral probiotics have been shown to be beneficial in preventing ventilator-assisted pneumonia in adults (83) and upper respiratory tract infections (84) but their benefits for respiratory health in neonates remain to be seen.

Bacterial metabolites such as short chain fatty acids (SCFA) may also be a mechanism for gut microbes influencing respiratory health (85). SCFA act directly on both epithelial cells and immune cells significantly affecting the immune response (86). Changes to the fiber content in the diet of mice affect the bacterial composition within the gut and levels of serum SCFA. Mice fed a high fiber diet had more Bacteroides in the gut and raised serum SCFA compared to mice fed a low fiber diet who had more Firmicutes and decreased SCFA. Raised SCFA was associated with reduced allergic airway inflammation (87).

The common mucosal response theory suggests that antigens presented from one mucosal surface can affect lymphoid cell migration to other mucosal sites, influencing immune responses at the distal site (88). The immunological mechanisms explaining this effect are summarized in Figure 1 (78). Bacterial derived antigens presented by dendritic cells (DCs) lead to T-cell activation within mucosa-associated lymphoid tissue. Tissue-specific T-cells expressing chemokine receptor 4 drawn to the lung along with the more generic chemokine receptor 6 (78). Differences in the gut microbiota will result in altered antigen presentation and T-cell activation. Oral probiotics could also influence respiratory health *via* this mechanism. DCs are critical to antigen presentation. Probiotics lead to DC secretion of the regulatory cytokines IL-10 and IL-12 leading to a shift in the T-helper cell population toward Th1 dominance (89). The improved pathogen clearance and reduction in the Th2 response provides a potential mechanism for beneficial effect of probiotics in the lung.

There may also be a lung–gut axis with the reverse processes in action. Exposing the lungs of mice to lipopolysaccharide results in changes to the bowel flora (90). The impact of acute lung injury and infection may not be restricted to the lungs. Further work is needed to verify these finding and explore any clinically relevant impact.

The gut–lung axis is an example of the potential influence of the gut microbiome over many body systems (91–93). A more detailed understanding of this process may influence feeding and probiotic strategies in neonatology.

**Figure 2:**
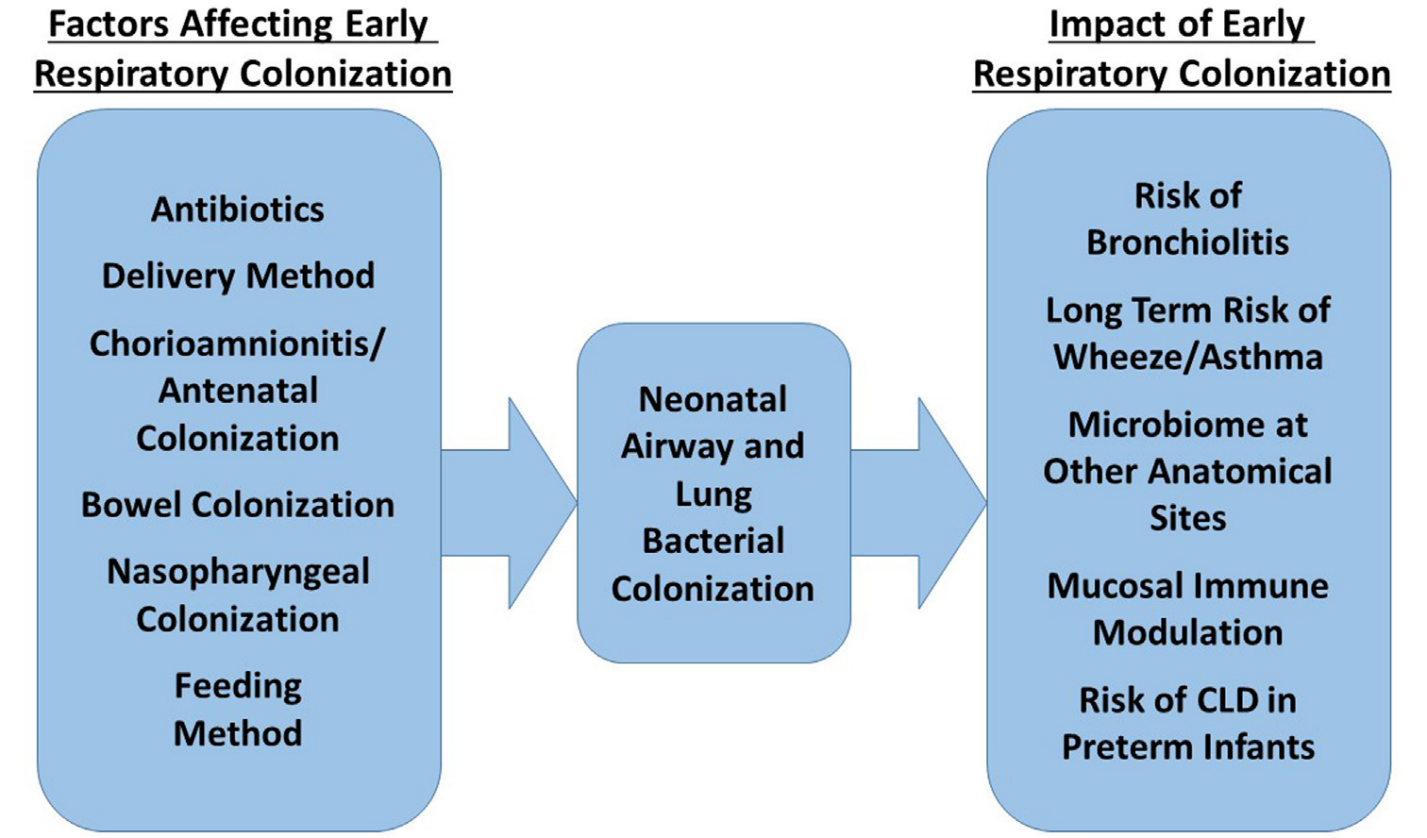
**Factors influencing the composition of the neonatal respiratory microbiome and possible impact of this microbiome on future health**.

Figure 2 summarizes the factors currently shown to affect neonatal bacterial respiratory colonization and the effects colonization patterns may have.

## The Future

### Identifying the Microbiota and Dysbiosis

Overcoming technical challenges in obtaining samples from the lower airways and lungs of preterm infants can potentially lead to the development of accurate biomarkers for accurately identifying lung microbial colonization. Certainly, molecular methods provide improved sensitivity to traditional culture-based techniques, but modern methods will need to separate infection from colonization, viable from non-viable and commensals from pathogenic bacteria. In clinical work, the transition from culture-based pathogen identification to molecular-based techniques is already underway with PCR tests for, among others, *Cytomegalovirus* and *Neisseria gonorrhoeae* used routinely (94). These techniques have already been applied to respiratory bacteria in samples from patients with cystic fibrosis for research purposes (95). It is likely that most routine clinical microbiology will be performed using molecular-based techniques in the future.

### Influencing the Microbiota

Probiotics and prebiotics are attempts to manipulate the microbiome of the gut to promote a healthy microbiome. This approach has received widespread support in the neonatal community and a favorable Cochrane review showing efficacy of probiotics in preventing NEC in preterm infants treated with *Lactobacillus* and/or *Bifidobacterium* species (96). Alternative strategies for gut microbiome alteration have been tried, with variable success, in adults using fecal transplantation in *Clostridium difficile* infection (97). Managing the human microbiome with antibiotics has been occurring for decades. The liberal use of antibiotics on neonatal units alters neonatal gut (56), and likely respiratory, microbiota significantly, with unknown long term effects. Routine use of azithromycin to treat possible *Ureaplasma* infection in pre-terms has been suggested as a way to reduce rates of CLD (98, 99). The impact this would have on other organisms at this important stage of early colonization is unknown and will be important to determine as part of any future trial.

Rising antibiotics resistance and further understanding of host–microbe interactions may lead to a change in emphasis from eradication of pathogens by antibiotics to out competing pathogenic organisms by restoring the healthy, premorbid microbiome. Dysbiosis in the lungs may perhaps be amenable to treatment with nebulized probiotics. Early mouse work has demonstrated nasal administration of probiotic bacteria can protect against respiratory infection (89). Furthermore, as we understand the source of lung colonizing bacteria and factors influencing the early colonizers, manipulation of bacterial acquisition may be possible.

Studying the gut–lung axis may provide significant insights into cultivating the optimal respiratory microbiome. Dietary changes are known to affect the gut microbiome (100). Breast milk is recommended for all infants for many good reasons including reduced upper respiratory tract infections (101). Part of the benefits of breast milk may be through manipulation of the gut and/or respiratory microbiota, but this needs further exploration.

Early microbial programing demonstrates the importance of a long-term view of all areas of neonatal care. Future studies focusing only on the neonatal period will not detect the effects of microbiological programing.

As the understanding of the role of the microbiome increases from observational studies, it is likely that interventional studies will seek to manipulate the respiratory microbiome using established and novel techniques. Detailed work to grasp the complex symbiosis of host and colonizers is needed to minimize the risks of these treatments.

## Conclusion

The proponents of microbiome research suggest understanding the microbiome could lead to highly individualized care based on a patients colonization patterns, preventing acute infections, and long-term disease risks by microbiome manipulation (102, 103). The respiratory tract lags behind the gut in terms of the understanding of the role of commensal bacteria in health and disease. In neonatal practice, further work is needed to understand the early colonization and how this can be optimized. The important window of opportunity for influencing long-term health through microbiome-mediated effects is likely to be in the neonatal period.

## Author Contributions

DG performed the literature search and wrote the manuscript. SK reviewed the manuscript and authorized the final version.

## Conflict of Interest Statement

The authors declare that the research was conducted in the absence of any commercial or financial relationships that could be construed as a potential conflict of interest.
